# Computational Modeling of In Vitro Swelling of Mitochondria: A Biophysical Approach

**DOI:** 10.3390/molecules23040783

**Published:** 2018-03-28

**Authors:** Vladimir I. Makarov, Igor Khmelinskii, Sabzali Javadov

**Affiliations:** 1 Department of Physics, University of Puerto Rico, Rio Piedras Campus, San Juan, PR 00931-3343, USA; vmvimakarov@gmail.com; 2 Faculty of Sciences and Technology, Department of Chemistry and Pharmacy, and Interdisciplinary Centre of Chemistry of Algarve, University of Algarve, 8005-139 Faro, Portugal; ikhmelin@ualg.pt; 3 Department of Physiology and Biophysics, University of Puerto Rico, Medical Sciences Campus, San Juan, PR 00936-5067, USA

**Keywords:** mitochondria, biophysical modeling, mitochondrial swelling, permeability transition pore, kinetic analysis, matrix volume, calcium

## Abstract

Swelling of mitochondria plays an important role in the pathogenesis of human diseases by stimulating mitochondria-mediated cell death through apoptosis, necrosis, and autophagy. Changes in the permeability of the inner mitochondrial membrane (IMM) of ions and other substances induce an increase in the colloid osmotic pressure, leading to matrix swelling. Modeling of mitochondrial swelling is important for simulation and prediction of in vivo events in the cell during oxidative and energy stress. In the present study, we developed a computational model that describes the mechanism of mitochondrial swelling based on osmosis, the rigidity of the IMM, and dynamics of ionic/neutral species. The model describes a new biophysical approach to swelling dynamics, where osmotic pressure created in the matrix is compensated for by the rigidity of the IMM, i.e., osmotic pressure induces membrane deformation, which compensates for the osmotic pressure effect. Thus, the effect is linear and reversible at small membrane deformations, allowing the membrane to restore its normal form. On the other hand, the membrane rigidity drops to zero at large deformations, and the swelling becomes irreversible. As a result, an increased number of dysfunctional mitochondria can activate mitophagy and initiate cell death. Numerical modeling analysis produced results that reasonably describe the experimental data reported earlier.

## 1. Introduction

Mitochondria are widely accepted as the powerhouse and provide up to 90% of the ATP necessary for the cell. They are also important for the regulation of ionic homeostasis, cell growth, redox signaling, and cell death [1,2,3]. The electron transport chain (ETC) generates an electrochemical gradient across the inner mitochondrial membrane (IMM). This gradient, induced by the mitochondrial membrane potential (ΔΨ_m_) and the H^+^ gradient, is known as the proton-motive force that drives ATP synthesis. Mitochondria contain two membranes, the outer mitochondrial membrane (OMM) and the IMM, with an intermembrane space (IMS) between them. The OMM is a semi-permeable membrane allowing the passage of species with a molecular weight up to 6 kDa. The IMM is impermeable under normal aerobic conditions and transports selected ions and solutes through specific channels and exchangers.

**Figure 1:**
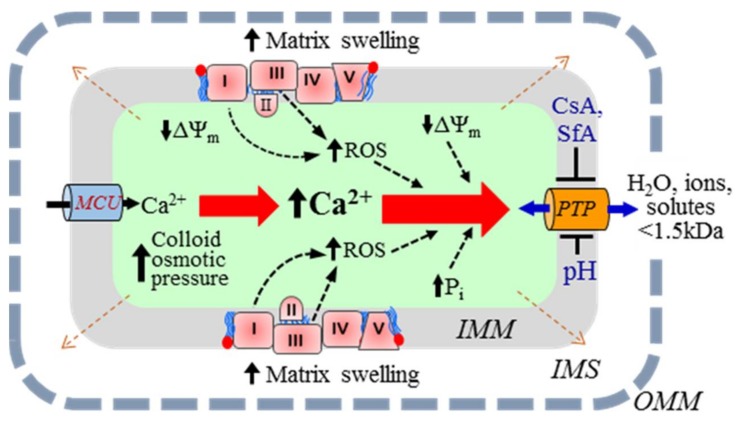
The mechanism of Ca^2+^-induced swelling of mitochondria. (See text for details.)

An increase in mitochondrial Ca^2+^ and reactive oxygen species (ROS) in response to oxidative and energetic stresses stimulates mitochondria-mediated cell death. Also, proteolysis due to protein oxidation can alter the structural and functional integrity of the IMM. The main mechanism of mitochondria-mediated cell death involves excessive matrix swelling induced by the opening of the non-selective channels, permeability transition pores (PTP) in the IMM [4,5,6] (Figure 1). The detailed molecular structure of PTPs is yet unknown. Although early studies revealed adenine nucleotide translocase (ANT) and voltage-dependent anion channel (VDAC) as the core components of the PTP complex, subsequent genetic studies noted a regulatory rather than structural role to these proteins in PTP formation. Cyclophilin D has been broadly accepted as a major regulator of the PTP (*reviewed in* [7]). Studies from several groups suggested that F_1_F_O_-ATPase was involved in the PTP complex [8,9]; however, recent findings challenged the pore-forming role of F_1_F_O_-ATPase [10,11].

The dynamics of PTP opening are usually described using various phenomenological approaches. The PTP opening can occur in the low-conductance (reversible) or high-conductance (irreversible) mode [6,12,13]. Low-conductance PTP flickering creates permeability to solutes up to 300 Da, mostly ions, and induces negligible matrix swelling [14]. However, the opening of the low-conductance PTP can initiate IMM depolarization [15]. Notably, the low-conductance PTP induction can regulate ATP synthesis through activation/inhibition of the Krebs cycle by Ca^2+^ in the matrix [16,17]. Furthermore, mitochondria are sensitive to small changes in the matrix volume that may be regulated by the low-conductance PTP. Increases in the matrix volume within the physiological range stimulate the ETC activity, ATP production, fatty acid oxidation, and other metabolic pathways [18]. The high-conductance open-state PTP has a channel ~3 nm in diameter, allowing for the diffusion of all species up to 1.5 kDa [4,19,20]. Therefore, PTP opening stimulates the free bi-directional movement of low molecular weight species (water and solutes) across the IMM, while the indiffusible proteins remain in the matrix. Consequently, an increase in the colloid osmotic pressure causes matrix swelling. Due to the ensuing OMM rupture, apoptotic proteins (e.g., cytochrome *c*) are released into the cytosol, promoting cell death via apoptosis [21,22]. It should be noted that whether cell death occurs through apoptosis or necrosis depends on the ATP level. Since apoptosis is the ATP-dependent process, at low (<50%) ATP, despite release of apoptotic proteins from the IMS, the cell will apparently die by necrosis rather than apoptosis.

Calcium is a key inducer of both PTP-dependent and PTP-independent swelling of mitochondria (Figure 1). Cytoplasmic Ca^2+^ is rapidly taken up by mitochondria due to the proton-motive force generated by the ETC [23]. The mitochondrial calcium uniporter (MCU) is the main channel that transports Ca^2+^ to the matrix when PTPs are closed. In fact, the MCU is a low-affinity and high-capacity channel that mediates Ca^2+^ influx in a ΔΨ_m_-dependent manner [24]. Mitochondrial Ca^2+^ efflux occurs in three ways: (i) the mitochondrial Na^+^/Ca^2+^ exchanger, the main contributor, most relevant in muscle mitochondria [25]; (ii) the H^+^/Ca^2+^ antiporter, an Na-independent pathway [20,26,27]; and (iii) opening of the PTPs. The PTP opening occurs due to Ca^2+^ overload, inducing swelling of mitochondria caused by the influx of water and ions through the open pores [25,28].

In addition to Ca^2+^, H^+^ also plays an important regulatory role in PTP induction and, thus, mitochondrial swelling. Likely, H^+^ regulates the sensitivity of the PTP towards Ca^2+^; low pH inhibits pore opening, whereas pH above 7.0 stimulates permeability transition [26,29]. High H^+^ levels have been shown to inhibit the PTP induction through cyclophilin D [30] and F-ATP synthase [31]. Therefore, pharmacological agents that reduce pH and create temporary acidosis are able to prevent PTP opening and exert beneficial effects in pathological conditions associated with mitochondrial Ca^2+^ overload (e.g., ischemia–reperfusion) [32,33].

Modeling of mitochondrial swelling is important for the simulation and prediction of in vivo events under physiological and pathological conditions. The lack of knowledge on the molecular identity of the PTP complex obscures the understanding of the physical and chemical mechanisms of pore opening and mitochondrial swelling. Previous studies proposed several kinetic approaches to developing an optimized model of mitochondrial swelling. However, they do not provide an adequate mechanism underlying the transition from a reversible to an irreversible swelling state and should be improved by including additional factors. In particular, previous models did not take into consideration ΔΨ_m_, the major parameter that regulates mitochondrial PTP induction, swelling, and cell death [34,35,36]. Simultaneous analysis of swelling and the dynamics of ΔΨ_m_ loss are important for understanding the relationship between changes in the matrix volume, ion flux, and mitochondrial bioenergetics. Also, previous models used the kinetic limit for the analysis of the transport of ions and solutes. As a result, diffusion was assumed to be faster than the respective kinetic rates. Therefore, an improved model should include diffusion-limited transport of Ca^2+^ from the cytosol to the matrix to generalize the modeling approaches. 

We have earlier reported [37] a simple model to interpret the experimental data on Ca^2+^-induced mitochondrial swelling. In the current study, we further develop modeling analysis including the transport dynamics of different ions and species across the IMM, and their effects on matrix metabolism, with the objective to describe mitochondrial swelling based on detailed physical and chemical characteristics. Our model describes the mechanism of transition from reversible to irreversible swelling in mitochondria. We developed a FORTRAN code to carry out numerical simulations, with varying values of the model parameters. Our modeling approach is based on the experimental data reported earlier by different authors. It includes the mechanism of matrix swelling, where the IMM rigidity is described by the rigidity tensor, its components dependent on the IMM deformation scale. Simultaneously, the osmotic pressure in the matrix is compensated for by the IMM deformation. Based on earlier suggestions [38], our model couples the PTP opening dynamics with the matrix pH. We conclude that the model is suitable for the analysis of the transition of mitochondria from the reversible to irreversible swelling. However, the applicability range of the model is quite limited, as it only takes into account Ca^2+^, K^+^, and H^+^ to describe the irreversible swelling. In future, we plan to include a description of irreversible swelling in the more complex models with all of the relevant species, which currently disregard the possibility of irreversible swelling.

## 2. Model Description

There are two different approaches to the modeling of mitochondrial swelling dynamics [39]. The first approach introduces phenomenological kinetic parameters, which are fitted for better reproduction of the experimental kinetic results. The second approach develops a biophysical model based on the ionic and hydrodynamic fluxes across the IMM, and changes in ΔΨ_m_ and mitochondrial respiration rates [19,40].

The role of Ca^2+^ in mitochondrial swelling has been assessed using a simple kinetic approach in rat liver mitochondria [41]. The kinetics of the permeability transition were analyzed within a model based on the assumption that the transition follows first-order kinetics and that the solute diffusion rate depends on the pore conformation. In addition, several theoretical studies concerning mitochondrial swelling dynamics were reported [39,42,43,44,45,46]. Another study reported a model of mitochondrial ion transport and its application to the analysis of different modes of Ca^2+^ uptake by mitochondria [47]. However, this model failed to reproduce the swelling and the role of PTP opening in swelling. Likewise, several systematic studies reported modeling of Ca^2+^ transport across the IMM, and also PTP induction [42,43,44,45]. Their approach is based on a kinetic step that may be presented as Ca^2+^*_out_* ↔ Ca^2+^*_in_*, describing Ca^2+^ ion exchange between the outside and inside of the IMM.

### 2.1. A Basic Model of Mitochondrial Dynamics

**Figure 2:**
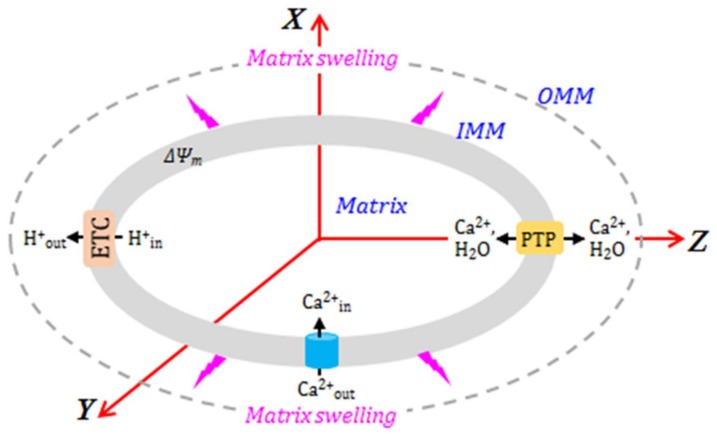
Biophysical approach to modeling mitochondrial swelling.

A simple scheme of the proposed model of Ca^2+^-induced mitochondrial swelling is shown in Figure 2. This model is an extended version of our earlier approach [37]. The Ca^2+^ concentration affects ΔΨ*_m_* = Ψ*_m, out_* – ΔΨ*_m, in_*, the proton concentration in the matrix *C_H, in_*, the PTP opening dynamics, described by the *P_PTP, op_* parameter, matrix concentration of the respiration activator (A), matrix osmotic pressure *P_os_* and matrix swelling. Mitochondrial swelling is induced by ionic/neutral species transport in/out of the matrix, which creates osmotic pressure inside the matrix. The osmotic pressure is compensated for by the IMM deformation induced by swelling. The present modeling approach aims to reproduce the IMM swelling dynamics, which is included in the transport model for the ionic and neutral species going in or out of the matrix, along with PTP opening dynamics.

We used the Goldman equation to describe ionic species transport in and out of the matrix [47], which may be presented as follows:

(1) $$J_{i}^{}=J_{i}^{out}-J_{i}^{in}=p_{i}\frac{k_{B}T\Delta \Psi_{m}}{z_{i}\left|e\right|}\frac{C_{i}^{in}e^{-\frac{z_{i}\left|e\right|\Delta \Psi_{m}}{k_{B}T}}-C_{i}^{out}}{1-e^{-\frac{z_{i}\left|e\right|\Delta \Psi_{m}}{k_{B}T}}},$$

where *J_i_* is the flux of *i*-th ionic species, *J_i_^in^* and *J_i_^out^* are the *i*-th ionic species fluxes coming in and going out of the matrix, *k_B_* is the Boltzmann constant, *T* is the absolute temperature, *z_i_* is the relative ionic charge, |*e*| is the absolute electron charge, $C_{i}^{in}$ and $C_{i}^{out}$ are the concentrations of the *i*-th ionic species inside and outside the matrix, respectively, and *p_i_* is the reduced permeability of the IMM to the *i*-th ionic species. The transport of neutral species may also be described by Equation (1), substituting ΔΨ*_m_* → 0, i.e.,

(2) $$J_{i}^{}=J_{i}^{out}-J_{i}^{in}=p_{i}^{\left(0\right)}\left(C_{i}^{in}-C_{i}^{out}\right),$$

where *p_i_*^(0*)*^ is the diffusion coefficient of the respective species in the uniport channels. We assumed *p_i_*^(0*)*^ = 0 for the uniport of the ionic species. Our model uses both Equations (1) and (2). We used published values of the model parameters in our numerical analysis, with the respective data and references listed in Table 1.

### 2.2. Ca^2+^ Transport across the IMM

Calcium influx through the IMM may be described by Equation (1), taking into account its background cytosolic concentration *C*_0,*cb*_ = 0.5 μM [48]. The effective reduced permeability of Ca^2+^ uniport across the IMM is listed in Table 1. Our model includes the transport of Ca^2+^ only. The IMM is impermeable to Ca^2+^, K^+^, Na^+^, H^+^ and other ions; specific channels and exchangers control their flux and concentrations in the mitochondrial matrix. Specifically, mitochondrial K^+^ balance is controlled by ATP-dependent (mitochondrial K_ATP_ channel) and Ca^2+^-dependent channels responsible for influx, and by K^+^/H^+^ exchanger responsible for removal of the excess matrix K^+^. Sodium balance is controlled by Na^+^/Ca^2+^ (influx) and Na^+^/H^+^ (efflux) exchangers [20]. As we already stated, the present model does not include Na^+^ influx/efflux mechanisms. Therefore, the respective processes were omitted. However, we included the K^+^/H^+^ exchange mechanism because K^+^ transport at low [Ca^2+^] (low-conductance PTP) induces oscillations of the mitochondrial volume [53,54]. This effect resulting from K^+^ transport across the IMM is appropriately described by modified Equation (1), which may be presented as follows:

(3) $$\begin{array}{l} J_{K^{+}}^{K/H}=J_{K^{+}}^{out}-J_{K^{+}}^{in}=p_{K^{+},H^{+}}\frac{k_{B}T\Delta \Psi_{m}}{\left|e\right|}\frac{C_{K^{+}}^{in}e^{-\frac{\left|e\right|\Delta \Psi_{m}}{k_{B}T}}-C_{K^{+}}^{out}}{1-e^{-\frac{\left|e\right|\Delta \Psi_{m}}{k_{B}T}}}\\ J_{H^{+}}^{H/K}=J_{H^{+}}^{out}-J_{H^{+}}^{in}=p_{H^{+},K^{+}}\frac{k_{B}T\Delta \Psi_{m}}{\left|e\right|}\frac{C_{H^{+}}^{in}e^{-\frac{\left|e\right|\Delta \Psi_{m}}{k_{B}T}}-C_{H^{+}}^{out}}{1-e^{-\frac{\left|e\right|\Delta \Psi_{m}}{k_{B}T}}} \end{array},$$

where the permeability for the K^+^ and H^+^ ion transport through the membrane depends linearly on H^+^ and K^+^ concentrations: $p_{K^{+},H^{+}}=p_{K^{+},H^{+}}^{\left(0\right)}\left|C_{H^{+}}^{out}-C_{H^{+}}^{in}\right|$ and $p_{H^{+},K^{+}}=p_{H^{+},K^{+}}^{\left(0\right)}\left|C_{K^{+}}^{out}-C_{K^{+}}^{in}\right|$, respectively. The values of the $p_{K^{+},H^{+}}^{\left(0\right)}$ and $p_{H^{+},K^{+}}^{\left(0\right)}$ parameters are listed in Table 1.

### 2.3. PTP Transport of Ionic and Neutral Species

As we already mentioned, PTP opening also controls transport of different ions and neutral species to and from IMM. Factors controlling PTP opening include changes in [Ca^2+^] and ΔΨ_m_, and matrix pH dynamics [48]. Hence, for simplicity, we assume that Ca^2+^ affects the PTP opening dynamics only indirectly, by inducing changes in ΔΨ_m_ or matrix pH. Presently, we follow the modeling approach proposed earlier [38], adding the model component required to describe the mitochondrial swelling dynamics. According to this approach, we assume that ΔΨ_m_ has no direct influence on the PTP opening dynamics in the autoinduced release of mitochondrial Ca^2+^. It is known that PTPs are completely open at pH ≥ 7.3–7.5, and completely closed at pH ≤ 7.0 [48]. Based on these facts, we define the probability of PTP opening as follows:

(4) $$P_{PTP}=\frac{\alpha {\left(pH-7.0\right)}^{n}}{1+\alpha {\left(pH-7.0\right)}^{n}}.$$

**Figure 3:**
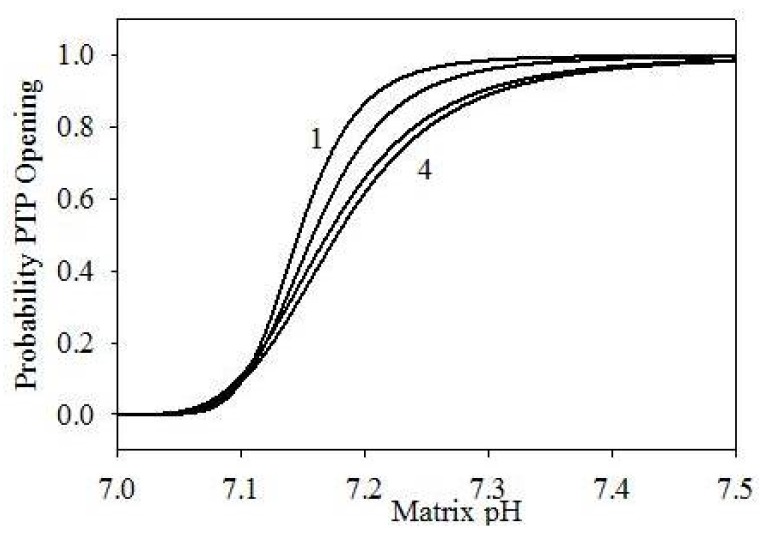
PTP opening probability in function of matrix pH, using different parametrizations: (1) *α* = 10^5^, *n* = 6; (2) *α* = 10^4^, *n* = 5; (3) *α* = 1.2 × 10^3^, *n* = 4; (4) *α* = 10^3^, *n* = 4.

This expression correctly produces *P_PTP_* = 0 at pH < 7.0, and *P_PTP_* = 1 at pH > 7.5. Figure 3 shows the probability dependence on pH at different values of *α* and *n*. On the other hand, the PTP opening rate and the PTP closing rate may be presented as follows:

$$\begin{array}{l} W_{op}^{PTP}=k_{op}n_{PTP,cl}^{m}\\ W_{cl}^{PTP}=k_{cl}n_{PTP,op}^{m}\\ n_{PTP,cl}^{m}+n_{PTP,op}^{m}=n_{PTP}^{m} \end{array},$$

where *k_i_* is the respective rate constants (min^−1^), $n_{PTP}^{m}$ is the number of PTP on the IMM of a single mitochondrion, $n_{PTP,op}$ is the number of open PTP, and $n_{PTP,cl}$ is the number of closed PTP.

Using these notations, the equation for the PTP opening dynamics may be presented as follows:

(5) $$\begin{array}{l} \frac{dn_{PTP,op}}{dt}=k_{op}n_{PTP}^{m}-\left(k_{op}-k_{cl}\right)n_{PTP,op}^{m}\\ n_{PTP,op}^{m}\left(t\right)=\frac{k_{cl}n_{PTP}^{m}}{\left(k_{op}-k_{cl}\right)}\left(e^{-\left(k_{op}-k_{cl}\right)t}\right) \end{array}.$$

The latter relationship describes the dynamics of PTP opening. However, it does not include explicitly any effects of the matrix pH; these effects should then be included into the *k_i_* parameters. This makes the analysis quite complex. To simplify the calculations, we use Equation (3) instead of Equation (5). The matrix pH is now a time-dependent function, which we introduce below. Ion fluxes through the PTP may also be described by the modified Goldman Equation (1), and presented as follows:

(6) $$\begin{array}{l} J_{i,PTP}^{}=p_{i,PTP}P_{PTP}n_{PTP}^{m}\frac{k_{B}T\Delta \Psi_{m}}{z_{i}\left|e\right|}\frac{C_{i}^{in}e^{-\frac{z_{i}\left|e\right|\Delta \Psi_{m}}{k_{B}T}}-C_{i^{}}^{out}}{1-e^{-\frac{z_{i}\left|e\right|\Delta \Psi_{m}}{k_{B}T}}}. \end{array}$$

In case of H^+^ transport, both $C_{H+}^{in}$ and $C_{H+}^{out}$ are variable parameters. The permeability coefficient for H^+^ coming through PTP is unknown. We assume that its value is close to that for Ca^2+^, with both values listed in Table 1, where *p_H+,PTP_* = *p_Ca_*_2+_,*_PTP_*/2 due to the difference in ionic charges [55]. We also assume that the value of K^+^ permeability for PTP transport is the same as that for H^+^. Thus, we included H^+^, K^+^, and Ca^2+^ transport through PTP into the current model. In our calculations, we used the conservation relationships:

$$C_{K+}^{in}\;+\;C_{K+}^{out}\;=\;C_{K+,0}\;\mathrm{and}\;C_{Ca2+}^{in}\;+\;C_{Ca2+}^{out}\;=\;C_{Ca2+,0}$$

for K^+^ and Ca^2+^, and used a constant external hydrogen ion concentration outside of the matrix $C_{H+}^{out}$ (Table 2). We also used the same initial [H^+^] in the matrix in all of the simulation runs and recorded its evolution in time. In the case of depolarized IMM, we may represent the ion transport through the PTP by the relationship similar to Equation (2):

(7) $$J_{i}^{}=J_{i}^{out}-J_{i}^{in}=p_{i,PTP}^{\left(0\right)}P_{PTP}n_{PTP}^{m}\left(C_{i}^{in}-C_{i}^{out}\right),$$

where *p_i,PTP_*^(0*)*^ is the effective diffusion coefficient of the respective species through PTP, unknown for live cells. However, we neglect the thermal diffusion of ions through PTP in a depolarized IMM, Equation (7), as the ion release, in this case, is dominated by the hydrodynamic flux induced by mitochondrial rigidity.

### 2.4. Weak Acid Dissociation

Since the probability of PTP opening is directly dependent on the matrix [H^+^] (*C_H+_*), we include the dissociation equilibrium for a weak acid AH. We use the latter as a simplified model for the second dissociation of phosphoric acid, falling into the pH interval of interest with p*K*_a_ = 7.20. In this case, we obtain:

(8) $$C_{H^{+}}^{}=C_{A^{-}}^{}=\frac{1}{2}\left\{\sqrt{K_{AH}\left(K_{AH}+4C_{AH}^{\left(0\right)}\right)}-K_{AH}\right\},$$

where *K_AH_* is the equilibrium constant of the acid and $C_{AH}^{\left(0\right)}$ its initial concentration. Assuming that there are other sources of H^+^ producing its background concentration $C_{H+}^{bg}$, Equation (8) becomes:

(9) $$C_{H^{+}}^{}=C_{A^{-}}^{}=\frac{1}{2}\left\{\sqrt{{\left(K_{AH}+C_{AH}^{bg}\right)}^{2}+4K_{AH}C_{AH}^{\left(0\right)}}-\left(K_{AH}+C_{H^{+}}^{bg}\right)\right\}$$

Next, we substitute concentrations by activities in Equations (1)–(8):

(10) $$a_{i}=\gamma_{i}C_{i},$$

where the activity coefficient *γ_I_* is determined using the Debye–Hückel theory [56]:

(11) $$\ln\left(\gamma_{i}\right)=-\frac{\sqrt{2\pi }{\left|e\right|}^{5}z_{i}^{2}}{\sqrt{{\left(\varepsilon \varepsilon_{0}k_{B}T\right)}^{3}}}\sqrt{I},$$

where *I* is the ionic force, given by:

(12) $$I=\frac{1}{2}\sum_{}C_{i}z_{i}^{2}.$$

We calculated activity coefficients using Equation (11). The mechanism involving electrodiffusion of anions and H^+^ through PTP may be extended to the transport of other weak organic acids to/from the matrix through PTP. The respective kinetic model for a weak acid AH is described by the following reactions:

1. AH*_out_* ↔ A^−^*_out_* + H^+^*_out_*
2. A^−^*_out_* ↔ A^−^*_in_*
3. H^+^*_out_* ↔ H^+^*_in_*
4. A^−^*_in_* + H^+^*_in_* ↔ AH*_in_*

The dissociation constants of weak acids are known quite well [56]. We assume that the dissociation equilibriums, described by Reactions (1) and (4), are much faster than diffusion, described by Reactions (3) and (4). We use the same values of the equilibrium constants inside and outside the matrix. Taking into account the relations (6), and (9) to (12), we described AH transport through PTP. Our model runs use the permeability value for the PTP transport of A^−^ reported earlier [38] (see Table 1).

### 2.5. Effect of Respiration (ETC Activity) on H^+^ Generation

Taking into account that ΔΨ_m_ directly affects the respiration and depends on the ETC activity [46,47], we used *W_A_*, the generation rate of the respiration activator A, in our model in the following form [55]:

(13) $$W_{A}=k_{A}a_{A,0}\left(1-e^{-\frac{\Delta \Psi_{m}}{\delta \left(\Delta \Psi_{m}\right)}}\right),$$

where *k_A_* [min^−1^mM^−1^] is the effective respiration rate constant, *δ*(ΔΨ*_m_*) is a variable parameter and *a_A,_*_0_ is the activator activity at zero time. The respiration rate is constant at ΔΨ*_m_* = 200 mV [38]. This was achieved in the model by requiring that *W_A_* = 0.99*k_A_a_A,_*_0_ at ΔΨ*_m_* = 200 mV, which results in *δ*(ΔΨ*_m_*) = 43.42 mV, the value used in all model calculations. The respiration activator flux may be presented by the modified Equation (7):

(14) $$J_{A}^{}=p_{A,PTP}^{}P_{PTP}n_{PTP}^{m}a_{A},$$

with the *p_A,PTP_* value listed in Table 1. 

Mitochondrial respiration induces the formation of H^+^ in the IMS due to its ejection from the matrix by ETC complexes I, III, and IV. Our model does not include the detailed mechanism of H^+^ transport into the IMS. Therefore, it controls [H^+^] by its generation via an irreversible process:

A → H^+^ + S^−^,

with the S^−^ product not participating in any other reactions. We determined the H^+^ transport using Equation (6) and now, discuss the implementation of the effects of H^+^ on ΔΨ_m_. We calculated the rate of ETC-induced H^+^ generation:

(15) $$W_{H^{+},A}=k_{A,H^{+}}a_{A},$$

where *k_A,H+_* is the H^+^ formation rate constant in the respiration cycle.

### 2.6. Model Implementation

The dynamics of ΔΨ_m_ is determined by the matrix ion fluxes, calculated as explained above. Thus, the system of equations describing the evolution of the model in time may be written as:

(16) $$\begin{array}{l} \frac{d\left(\Delta \Psi_{m}\right)}{dt}=\frac{F}{C}\left[2J_{Ca^{2+}}+J_{A^{-}}+J_{H^{+}}+J_{K^{+}}\right]\\ J_{H^{+}}=J_{H^{+},PTP}^{}+\frac{W_{H^{+},A}^{}}{S_{m}}+J_{H^{+}}^{H/K} \end{array}$$

(17) $$\frac{da_{Ca^{2+}}^{in}}{dt}=\left(J_{Ca^{2+}}+J_{Ca^{2+},PTP}\right)S_{m}$$

(18) $$\frac{da_{H^{+}}^{in}}{dt}=\frac{S_{m}}{B}\left(-J_{H^{+}}-J_{AH}\right)$$

(19) $$\frac{da_{K^{+}}^{in}}{dt}=\left(J_{K^{+}}^{K/H}+J_{K^{+},PTP}\right)S_{m}$$

(20) $$\frac{dA_{in}}{dt}=\left(W_{A}-W_{H^{+},A}-J_{A}S_{m}\right),$$

where *F* is the Faraday constant, *C* is the IMM electric capacity, *a_i_* is the activity of the respective ion, *S_m_* the mitochondrial surface area, and *B* = 3 × 10^5^ [38]. Here we assume that the evolution of *P_PTP_*, Equation (3) and that of *W_A_*, Equation (13), occurs much faster than the processes presented in Equations (16)–(20). We calculated the IMM electric capacity using the relationship $C=\frac{S_{m}}{h}$, where *h* = 10 nm is the IMM thickness, used as a constant value in our analysis.

### 2.7. Mitochondrial Swelling Dynamics

We complement our model with the mitochondrial swelling induced by osmosis. Osmotic pressure results from the concentration differences of various species inside and outside the matrix, written as follows [51,57,58]:

(21) $$\Delta P_{os}=k_{B}TN\sum_{i}\left(a_{i}^{in}-a_{out}^{in}\right),$$

where *N* is Avogadro’s number. The higher osmotic pressure inside the matrix induces mitochondrial swelling, while the IMM deformation compensates for osmotic pressure up to a certain limit, maintaining equilibrium:

(22) $$\Delta P_{os}=\Delta P_{IMM},$$

where Δ*P_IMM_* is created by the IMM deformation. In the analysis of mitochondrial deformation, we assume that osmotic water transport in/out of the matrix is much faster than the rates of processes described by Equations (16)–(20). Our model calculations also assume ellipsoid shape for the mitochondrial matrix, with the IMM rigidity described by the second-order tensor:

(23) $$g=\left(\begin{array}{ccc} g_{xx} & g_{xy} & g_{xz}\\ g_{yx} & g_{yy} & g_{yz}\\ g_{zx} & g_{zy} & g_{zz} \end{array}\right),$$

with the matrix shape described by a prolate ellipsoid

$$\frac{1}{a^{2}}\left(x^{2}+y^{2}\right)+\frac{z^{2}}{c^{2}}=1,$$

where the rigidity tensor may be diagonalized in the reference system shown in Figure 2:

(24) $$g\prime =\left(\begin{array}{l} \begin{array}{ccc} {g^{\prime }}_{xx} & 0 & 0\\ 0 & {g^{\prime }}_{yy} & 0\\ 0 & 0 & {g^{\prime }}_{zz} \end{array} \end{array}\right).$$

Using the rigidity tensor, we express the pressure created by the IMM deformation:

(25) $$\Delta P_{IMM}=\frac{{g^{\prime }}_{xx}\Delta x\left(t\right)+{g^{\prime }}_{yy}\Delta y\left(t\right)+{g^{\prime }}_{zz}\Delta z\left(t\right)}{S_{m}\left(t\right)},$$

where

Δ*x*(*t*) *= x*(*t*) – *a*, Δ*y*(*t*) *= y*(*t*) – *a*, Δ*z*(*t*) *= z*(*t*) – *c* (26)

and

(27) $$\begin{array}{l} S_{m}\left(t\right)=2\pi {\left(a+\Delta x\right)}^{2}\left(1+\frac{c+\Delta z}{\left(a+\Delta x\right)f}\arcsin\left(f\right)\right)\\ f=\sqrt{1-{\left(\frac{a+\Delta x}{c+\Delta z}\right)}^{2}};\;a+\Delta x<c+\Delta z \end{array}.$$

The matrix volume changes and the resulting changes in the concentrations/activities are given by:

(28) $$\begin{array}{l} V_{m}\left(t\right)=\frac{4}{3}\pi {\left(a+\Delta x\right)}^{2}\left(c+\Delta z\right)\\ a_{i}^{in}=\frac{V_{m}\left(t-\Delta t\right)}{V_{v}\left(t\right)}a_{i}^{in} \end{array},$$

where Δ*t* is the time step used in the numerical integration of Equations (16)–(20).

To take into account the irreversibility of the mitochondrial swelling at large IMM deformations, the tensor components vanish at high deformations:

(29) $$\begin{array}{l} g_{xx}=g_{yy}=g_{9}=g_{00}\left(1-\frac{\beta_{0}\Delta r_{}^{n_{1}}}{1+\beta_{0}\Delta r_{}^{n_{1}}}\right)\\ r=x\;or\;y\\ g_{zz}=g_{zz,0}\left(1-\frac{\beta_{z}\Delta z_{}^{n_{1}}}{1+\beta_{z}z_{}^{n_{1}}}\right) \end{array},$$

where *g*_00_, *g_zz,_*_0_, *β*_0_, *β_z_*, and *n*_1_ are adjustable parameters. 

We also need to add to Equations (16)–(20) the terms describing the effluxes of the modeled species through PTP due to excess matrix pressure. According to Poiseuille’s law, the volume of homogeneous fluid escaping through a cylindrical tube is given by [44]:

(30) $$J_{0}=\frac{\pi r^{4}}{8\eta l}\Delta P_{IMM},$$

where *r* is the effective PTP radius, *η* is the viscosity of the matrix fluid, and *l* is the IMM thickness. We define the effective PTP radius as:

$$r=r_{0}P_{PTP},$$

where *r*_0_ ≤ 1.5 nm [26]. Thus, the hydrodynamic efflux of *i*-th species is given by:

(31) $$J_{i}=J_{0}a_{i}n_{PTP}^{m}.$$

Thus, our model calculations used Equations (16)–(20) with added hydrodynamic flux terms according to Equation (30). Assuming spherically symmetric mitochondria (*g_xx_* = *g_yy_* = *g_zz_*), the equations describing mitochondrial swelling dynamics become much simpler. The volume change rate will be given by:

(32) $$\frac{d\Delta V}{dt}=\eta \Delta P_{os}-\kappa \Delta V,$$

where *η* is the coefficient defining the rate of water transfer inside the IMM, and *κ* the coefficient describing the water transfer outside the IMM due to rigidity, vanishing at high deformations:

(33) $$\kappa =\kappa_{0}\left(1-\frac{\gamma \Delta V_{}^{m}}{1+\gamma \Delta V_{}^{m}}\right).$$

Here, *κ*_0_ is the linear rigidity coefficient, and *γ* and *m* the parameters characterizing membrane properties. At small volume changes, Equation (32) becomes:

(34) $$\frac{d\Delta V}{dt}=\eta \Delta P_{os}-\kappa_{0}\Delta V.$$

Its solution is still quite complex, because Δ*P_os_* is a time-dependent function, including ion transport and dilution dynamics created by volume dynamics. Using a linear approximation to Equation (33), the latter equation may be solved using Equation (22), with the result given by:

(35) $$V(t)=\frac{\eta }{\kappa }\Delta P_{os}\left(1-e^{-\kappa t}\right).$$

Assuming *κ*^−1^ is much shorter than the characteristic times of other processes included in the model, Equation (34) takes the form:

(36) $$\Delta V=\frac{\eta \Delta P_{os}}{\kappa_{0}}.$$

The earlier approach presented to mitochondrial swelling dynamics [46] is based on Equation (35). This approach, however, has limited utility, as swelling involves dilution of various components; additionally, Equation (35) is only applicable to spherically symmetric systems with homogeneous swelling. This equation also disregards the irreversibility of large deformations, introduced here by Equation (32). 

All of the above considerations apply to the analysis of mitochondrial volume dynamics as a function of different system parameters. Our main objective was to gain an understanding of irreversible mitochondrial swelling, testing the new modeling approach. We are considering a set of equivalent mitochondria, with their number density given by *n_mit_* (cm^−3^), an additional model parameter. 

## 3. Numerical Experiments

Since mitochondrial dimensions vary significantly between biological species and cell types, our model analysis specifically targets mitochondria of the adult rat cardiomyocytes. We implemented the numerical model in a homemade FORTRAN code, with the input parameter values listed in Table 2.

Our numerical experiments used constant values for the *α*, *n*, *C_AH_*_,0_, *δ*(ΔΨ*_m_*), *C_A_*_,0_, *k_A_*, and *k_A_*_,*H+*_ parameters, selected (Table 2) according to previous reports [38,43,46,51,55,57,58,59,60]. We estimated the values of *g*_00_ and *g_zz,_*_0_ for spherical mitochondria based on their average mass and expected oscillation frequency [61]. We used the experimental criterion of irreversible swelling observed upon 50% increase in volume; this translates into Δ*r* = 0.144*a* and Δ*z* = 0.144*c*, and the rigidity tensor components vanishing at *a* = 0.5 μm and *c* = 1 μm [61]. Thus, and using Equation (28), we selected *β*_0_, *β_z_*, and *n*_1_ values as listed in Table 2. We also introduced an outside [H^+^] = $C_{H+}^{out}$, kept constant during the model run. We also kept constant $C_{H+,0}^{in}$*^in^*, the matrix [H^+^], in each of the numerical experiments. The values of these two parameters were selected using previous reports [61], with the respective data listed in Table 2. We selected K^+^ and Ca^2+^ concentrations, which we varied independently, to cover their entire natural range. 

We solved the system of equations defining our model using a homemade FORTRAN code, and the parameters of Table 2, producing the following output parameters:

*φ* = ΔΨ*_m_*(*t*), mV,

*χ* = pH = −log([H^+^]*_in_*),

*η* = [Ca^2+^]*_in_*, μM,

*ξ* = [K^+^]*_in_*, μM,

with PTP opening probability *P_PTP_* and

*ζ = V_m_*(*t*)/*V_m_*(*t* = 0) – 1.

All of the calculations used ΔΨ*_m_*(*t* = 0) = 200 mV, $C_{Ca2+}^{in}$(*t* = 0) = 0.5 μM, and $C_{K+}^{in}$(*t* = 0) = 0. In all of the numerical experiments, we kept the ratio of the cell volume to the initial mitochondrial volume constant. Usually an adult rat cardiomyocyte contains ~5000 mitochondria, with the cell to mitochondrial volume ratio of ca. 2.50–2.86 [62]. Thus, the free cell volume per each mitochondrion is (2.50 − 2.86)*V_m_*(*t* = 0). Our numerical analysis additionally used the values of various constants as reported earlier [52,63,64] (Table 1).

## 4. Results and Discussion

**Figure 4:**
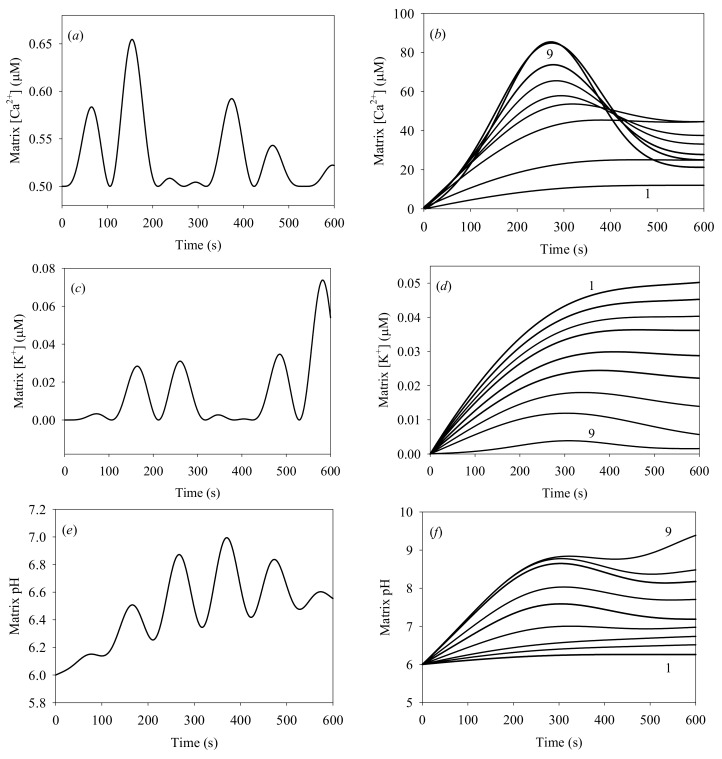
Time evolution of matrix [Ca^2+^]: (**a**) initial [Ca^2+^]*_out_* = 1.0 μM; (**b**) plots 1 to 9: initial [Ca^2+^]*_out_* = 25, 50, 75, 100, 150, 200, 300, 400, 500 μM; (**c**,**d**) evolution of the matrix K^+^ concentration versus initial cytosolic [Ca^2+^], for the same initial [Ca^2+^]*_out_* values; (**e**,**f**) evolution of the matrix pH versus initial cytosolic [Ca^2+^], for the same initial [Ca^2+^]*_out_* values. All calculations were carried out for the background [Ca^2+^]*_in_* = 0.5 μM, initial cytosolic [K^+^] = 0.1 μM, initial matrix pH = 6. The cytosolic pH = 7.0 was kept constant.

**Figure 5:**
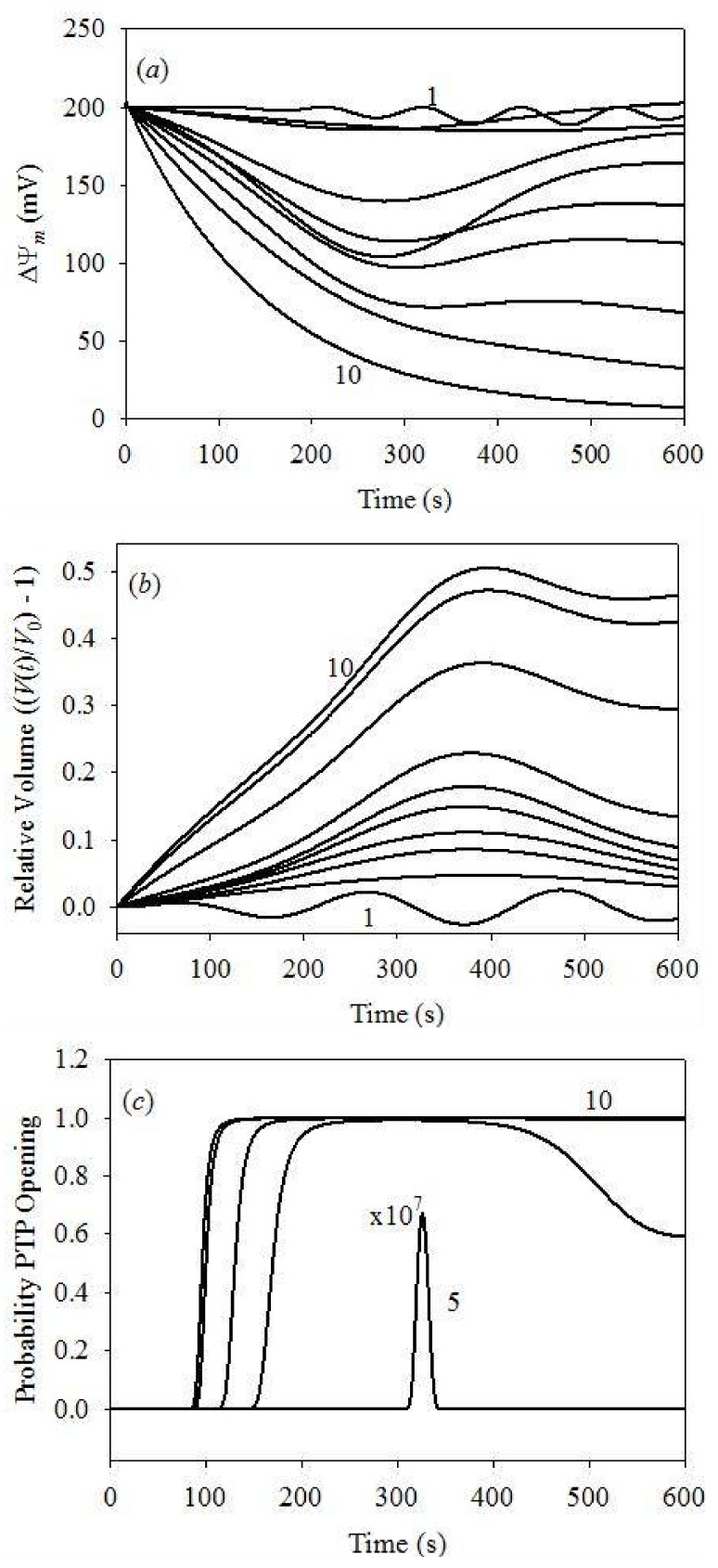
Dynamics of the IMM potential, mitochondrial volume and PTP opening probability for different initial cytosolic [Ca^2+^] at fixed initial cytosolic [K^+^] = 0.1 μM. (**a**) IMM potential dynamics: plots 1–10 for [Ca^2+^] = 1–500 μM; (**b**) the *V*(*t*)/*V*_0_–1 value at different cytosolic [Ca^2+^]: plots 1–10 for [Ca^2+^] = 1–500 μM; (**c**) dynamics of PTP opening probability: plot 5 for [Ca^2+^] = 100 μM is expanded by 10^7^; plots 6 to 10: [Ca^2+^] = 150–500 μM, respectively. *P_PTP_* = 0 at all times for [Ca^2+^] = 1, 25, 50 and 75 μM.

According to Table 2, we varied only two variable parameters: the initial $K_{out}^{+}$ and $Ca_{out}^{2+}$ concentrations. We made a total of 100 independent numerical experiments with the two concentrations of K^+^ and Ca^2+^ that were varied independently. However, we limited the analysis to only two concentrations of K^+^*_out_* (0.01 and 10 mM) and 10 different Ca^2+^*_out_* concentrations (Table 2), producing a total of the 20 most interesting numerical experiments. We calculated the dynamics of the $H_{in}^{+}$, $K_{in}^{+}$, and $Ca_{in}^{2+}$ concentrations, ΔΨ_m_, the probability of PTP opening, and the mitochondrial volume. The results of the initial cytosolic [K^+^] of 0.1 μM are plotted in Figure 4 and Figure 5.

Figure 4a,c,e show oscillations with ca. 97 s period of the Ca^2+^, K^+^, and H^+^ concentrations, for the initial cytosolic concentrations of Ca^2+^ and K^+^ of 1.0 and 0.1 μM, respectively. These oscillations result from heterogeneous processes developing at the IMM surface. Such oscillations occur only at low K^+^ and Ca^2+^ concentrations; they were detected experimentally [49,50], and described theoretically [38] in previous studies. Experimental measurements of [K^+^] oscillations and a theoretical analysis of oscillations were reported earlier, with oscillation periods of 20 s and 93 s [38,46].

These results are quite similar to those produced by our model, in particular for pH oscillations. Notably, our model differs from previous studies since it includes the swelling of mitochondria.

Figure 4b,d,f plot matrix [Ca^2+^], [K^+^] and matrix pH for different initial cytosolic [Ca^2+^]. An interesting result is observed at *t* < 600 s: the matrix [Ca^2+^] never exceeds 100 μM, while the initial cytosolic [Ca^2+^] is 500 μM. The model generates correct distribution between the “in” and “out” Ca^2+^ at low cytosolic [Ca^2+^] < 200 μM, in agreement with earlier reports [24,43]. However, strong deviations from the expected equilibrium distribution between “in” and “out” Ca^2+^ are produced at high cytosolic Ca^2+^ concentrations, between 300 and 500 μM. These deviations may be attributed to the PTP efflux of Ca^2+^ from the matrix, generated by the excess pressure within the matrix due to IMM deformation. This effect has not been reported before. All of the data plotted in Figure 4b,d,f also demonstrate that irreversible processes arise at initial cytosolic [Ca^2+^] in the 200–300 μM range. This result agrees with acceptable accuracy with the earlier reported experimental data [37,41].

Figure 5 demonstrates time evolution plots of *P_PTP_,* ΔΨ*_m_* and $\frac{V\left(t\right)}{V_{0}}-1$ in function of the initial [Ca^2+^] at a fixed initial cytosolic [K^+^] of 0.1 μM. Figure 5a plots the probability of PTP opening, showing that PTP begin to open only at the initial cytosolic [Ca^2+^] exceeding 100 μM. At this Ca^2+^ concentration, the probability of PTP opening is very small and limited in time. It increases with [Ca^2+^], reaching unity at 200 μM, although still for a short time only. Complete PTP opening was already observed at 150 s for [Ca^2+^] = 300 μM, and at 100 s for [Ca^2+^] ≥ 400 μM. Such PTP opening dynamics coincides with an acceptable level of accuracy with the data reported earlier [38,41].

Figure 5b plots the dynamics of ΔΨ_m_, with weak oscillations at the initial cytosolic [Ca^2+^] of 1.0 μM. This result agrees with earlier model data [38], reporting weak oscillations of ΔΨ_m_ in similar conditions. However, previous studies demonstrated different results reporting deep modulation of ΔΨ_m_, from 0 to 175 mV [46]. We will address these discrepancies in a future publication by the inclusion of the swelling dynamics. It should be noted that our swelling model is quite different, whereas the frequency of oscillation reported in our study is in agreement with the earlier reported model [46]. In conclusion, ΔΨ_m_ remains reversible for the initial cytosolic [Ca^2+^] in 25–200 μM range; however, it becomes irreversible at cytosolic [Ca^2+^] exceeding 300 μm, and completely depolarized at 500 μM. These results agree with the data reported earlier [26,43,65]. 

Figure 5c plots the dynamics of the matrix volume as $\frac{V\left(t\right)}{V_{0}}-1$ for clarity. Note that the volume oscillations appear already at the lowest cytosolic [Ca^2+^]. The same results were obtained earlier both in experimental measurements [54] and theoretical modeling analysis [46]. Once again, we found an acceptable agreement between the oscillation periods obtained here and reported earlier [46]. Apparently, reversible volume changes arise at initial cytosolic [Ca^2+^] < 300 μM, followed by irreversible swelling at high (>300 μM) [Ca^2+^]. The maximum volume increase of 50% of the initial mitochondrial volume was observed, as expected on the basis of the model parameterization. This volume increase corresponds to vanishing rigidity constant, Equation (28) and the data of Table 2, and, therefore, to the irreversible deformation and mitochondrial death. 

**Figure 6:**
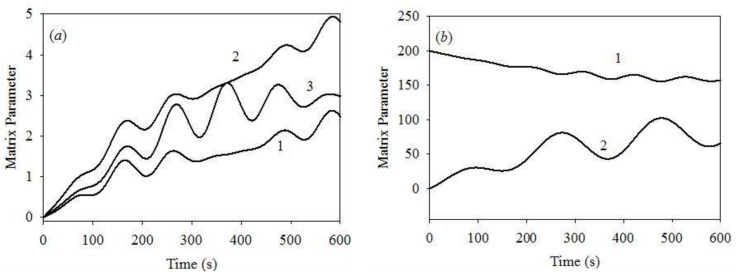
(**a**) Time evolution of matrix (1) [Ca^2+^] on the expanded scale $\left(\left(C_{Ca^{2+}}^{in}-0.5\right)\times 5\right)$, μM; (2) [K^+^], μM and (3) pH on the expanded scale ((pH−6)×5), obtained for the initial cytosolic [Ca^2+^] = 1.0 μM and [K^+^] = 10 μM; at these starting conditions *P_PTP_* = 0; (**b**) (1) ΔΨ_m_, mV; (2) mitochondrial volume V(t) on the expanded scale.

We report significant differences between the results obtained at [K^+^] of 0.1 and 10.0 μM, and [Ca^2+^] = 1.0 μM, see the plots of Figure 4a,c,e, Figure 5b and Figure 6, illustrating the dynamics of various system parameters. The results obtained at other [Ca^2+^] values are similar to those plotted in Figure 4 and Figure 5 at both [K^+^] values of 0.1 and 10.0 μM. We, therefore, only plot the results obtained for [K^+^] = 10 μM and [Ca^2+^] = 1 μM. Figure 6a shows that all of the matrix concentrations reveal weak oscillation dynamics, with periods similar to those obtained for the initial cytosolic [K^+^] = 0.1 μM. However, the modulation depth at [K^+^] = 10 μM is smaller than that obtained at the initial cytosolic [K^+^] = 0.1 μM. The same is apparent for the ΔΨ_m_ and mitochondrial volume dynamics. Thus, the changes in the initial cytosolic [K^+^] produce only minor changes in the dynamics of the key system parameters. 

Thus, our numerical model produces results that are different from those obtained in previous studies using a more sophisticated model [46]. The strongest discrepancies in the modulation depth of different model parameters appear at low [Ca^2+^]. For instance, we report ΔΨ_m_ modulation depth of 5–7% of the maximum value, while the previous study [46] reports 100% modulation depth, although still in the reversible mode. Apparently, even complete IMM depolarization does not affect the system dynamics in the respective model. This issue will be addressed in a future study, with the presently proposed swelling mechanism included in a more complex model. The swelling mechanism proposed in the previous report only considers the osmotic pressure, disregarding the interaction between the IMM rigidity and IMM deformation. 

## 5. Conclusions

In the present study, we implemented a simplified model of mitochondrial swelling that includes the contribution of osmosis and the IMM rigidity. This is a novel biophysical approach to mitochondrial swelling dynamics, which we will incorporate into more complex and sophisticated models. The numerical experiments in the framework of the developed model produced reasonable results, which describe the earlier reported experimental data with an acceptable accuracy.

## 6. Limitations of the Study

The modeling approach explored in the present study is limited by the simplified treatment of the biophysical and chemical processes, including transport of only three ionic species and a simplified respiration mechanism. However, we intentionally focused on the development of a more complex mechanism of mitochondrial swelling, and its usage in the analysis of the more sophisticated modeling approaches reported previously.

## Figures and Tables

**Table 1:** Parameter values used in modeling analysis.

Parameter	Definition	Value	Refs
*p*_Ca,UP_	Reduced permeability coefficient of the Ca^2+^ transport by uniporter	6.43 × 10^5^, min^−1^mV^−1^ (mg protein)^−1^ (K × esu/erg)	[18,48,49,50]
*p_K,H_*^(0)^	Reduced permeability coefficient of the K^+^/H^+^ exchange	3.20 × 10^5^, min^−1^mV^−1^ (mg protein)^−1^ (K × esu/erg) (μM_H_)^−1^	[47]
*P_H,K_*^(0)^	Reduced permeability coefficient of the H^+^/K^+^ exchange	3.20 × 10^5^, min^−1^mV^−1^(mg protein)^−1^ (K × esu/erg) (mM_K_)^−1^	[47]
*p_H+,PTP_*	Reduced permeability coefficient of the H^+^ transport by PTP	4.30 × 10^7^, min^−1^mV^−1^ (mg protein)^−1^ (K × esu/erg)	[47]
*p_Ca_*_2*+,PTP*_	Reduced permeability coefficient of the Ca^2+^ transport by PTP	8.61 × 10^7^, min^−1^mV^−1^ (mg protein)^−1^ (K × esu/erg)	[48]
*P_K+,PTP_*	Reduced permeability coefficient of the K^+^ transport by PTP	4.30 × 10^7^, min^−1^mV^−1^ (mg protein)^−1^ (K × esu/erg)	[51]
*p_A-,PTP_*	Reduced permeability coefficient of the A^−^ transport by PTP	6.43 × 10^2^, min^−1^mV^−1^ (mg protein)^−1^ (K × esu/erg)	[48]
*p_A,PTP_*	Reduced permeability coefficient of the A transport by PTP	32.1, min^−1^ (mg protein)^−1^	[48]
|*e*|	Absolute value of the electron charge	4.80286 × 10^−10^ esu	[52]
*k_B_*	Boltzmann constant	1.38044 × 10^−16^ erg/K	[52]
ΔΨ*_m_*	IMM potential	200 mV	[44,47,48]
*V_C_/V_m_*(*t* = 0)	Ratio of adult rat heart cell volume to total volume of all cell mitochondria	2.86	[49]

**Table 2:** Values of the input parameters.

*α*	1.2 × 10^3^									
*n*	4									
$C_{K+,0}^{out}$; μM	0.10	1.2	2.3	3.4	4.5	5.6	6.7	7.8	8.9	10
$C_{Ca2+,0}^{out}$; μM	1.0	25	50	75	100	150	200	300	400	500
−log($C_{H+,0}^{out}$)	7.00									
−log($C_{H+,0}^{in}$)	6.00									
$C_{AH,0}^{out}$; μM	50									
*δ*(ΔΨ*_m_*); mV	43.42									
$C_{A,0}^{in}$; μM	10									
*k_A_*; min^−1^	1									
*k_A,H+_*; min^−1^	1									
*g*_00_; dyn/nm	0.017									
*g_zz_*_,0_; dyn/nm	0.019									
*β*_0_; nm^−*n*1^	10^6^									
*β_z_*; nm^−*n*1^	10^7^									
*n*_1_	4									
